# Incidence and outcome of inappropriate in-hospital empiric antibiotics for severe infection: a systematic review and meta-analysis

**DOI:** 10.1186/s13054-015-0795-y

**Published:** 2015-02-16

**Authors:** Kristel Marquet, An Liesenborgs, Jochen Bergs, Arthur Vleugels, Neree Claes

**Affiliations:** Hasselt University, Faculty of Medicine and Life Sciences, Agoralaan, Building D, Room C53, Diepenbeek, BE3590 Belgium; Jessa Hospital, Stadsomvaart 11, Hasselt, BE3500 Belgium; Hasselt University, Faculty of Business Economics, Agoralaan, Building D, Diepenbeek, BE3590 Belgium; KU Leuven, Centre for Health Services and Nursing Research, Kapucijnenvoer 35/3, Leuven, BE3000 Belgium; Antwerp Management School, Health Care Management, Sint-Jacobsmarkt 9, Antwerp, BE2000 Belgium

## Abstract

**Introduction:**

The aims of this study were to explore the incidence of in-hospital inappropriate empiric antibiotic use in patients with severe infection and to identify its relationship with patient outcomes.

**Methods:**

Medline (from 2004 to 2014) was systematically searched by using predefined inclusion criteria. Reference lists of retrieved articles were screened for additional relevant studies. The systematic review included original articles reporting a quantitative measure of the association between the use of (in)appropriate empiric antibiotics in patients with severe in-hospital infections and their outcomes. A meta-analysis, using a random-effects model, was conducted to quantify the effect on mortality by using risk ratios.

**Results:**

In total, 27 individual articles fulfilled the inclusion criteria. The percentage of inappropriate empiric antibiotic use ranged from 14.1% to 78.9% (Q1-Q3: 28.1% to 57.8%); 13 of 27 studies (48.1%) described an incidence of 50% or more. A meta-analysis for 30-day mortality and in-hospital mortality showed risk ratios of 0.71 (95% confidence interval 0.62 to 0.82) and 0.67 (95% confidence interval 0.56 to 0.80), respectively. Studies with outcome parameter 28-day and 60-day mortality reported significantly (*P* ≤0.02) higher mortality rates in patients receiving inappropriate antibiotics. Two studies assessed the total costs, which were significantly higher in both studies (*P* ≤0.01).

**Conclusions:**

This systematic review with meta-analysis provides evidence that inappropriate use of empiric antibiotics increases 30-day and in-hospital mortality in patients with a severe infection.

**Electronic supplementary material:**

The online version of this article (doi:10.1186/s13054-015-0795-y) contains supplementary material, which is available to authorized users.

## Introduction

Infections are among the top three leading causes of death worldwide [1]. Septicaemia and pneumonia combined are the sixth most common causes of death in the United States [2]. Bloodstream infections (BSIs) are associated with substantial morbidity, mortality, and health-care costs [3]. Sepsis is one of the leading causes of death in the critically ill, with a mortality rate of 28% to 55% [4]. Antibiotics are the mainstay of treatment for these serious infections [5]. Antibiotic treatment for moderate to severe infections has to start early and, in the absence of evidence on the causative pathogen or its sensitivity to antibiotics, is often guided by empirical evidence [6].

Estimates of the potential benefit of appropriate empirical antibiotic treatment (AAT) vary widely [7-11]. Studies on the effect of inappropriate empiric antibiotic therapy (IAAT) on patient outcomes have yielded variable results [6,12]. Nevertheless, it is common wisdom that IAAT may lead to progressive deterioration and the development of complications or mortality [13-18].

Given the high incidence of infections and the not well-established relationship between empiric (I)AAT and clinical outcome [19-22], it is necessary to synthese the best available evidence. This systematic review with meta-analysis was conducted to synthesize the best available evidence regarding (1) the definition, (2) the incidence, and (3) the outcome of empiric IAAT.

## Methods

### Data sources and search strategy

Quantitative studies on the association between the use of empiric (I)AAT in patients with a severe infection and their outcome in public or private general hospital settings were searched in Medline. Studies published in the last 10 years (20 August 2004 to 20 August 2014) were selected as critical illness management changes continuously and earlier and earlier studies may be less relevant for current practice. The following Medical Subject Headings (MeSH) search terms and free-text terms were used either individually or in combination: ‘antibiotic’, ‘infection’, ‘appropriate’, ‘inappropriate’, ‘adequate’, ‘inadequate’, ‘outcome’, ‘mortality’, ‘survival rate’, ‘cost’, and ‘length of stay’. Only studies published in English, Dutch, German, or French were included. Reference lists of retrieved articles were hand-searched for additional relevant studies. A detailed description of the search strategy is included in the Additional file 1: digital content.

### Eligibility criteria

#### Study design

Potentially included study designs included randomized controlled trials (RCTs), non-randomized controlled trials, controlled before-after studies, interrupted time series, and repeated measures studies. Only studies reporting a quantitative evaluation regarding the association between the use of AAT or IAAT in patients with a severe infection and their outcomes within the hospital setting were included. The studies use (I)AAT as the independent variable and outcome—measured as mortality, hospital length of stay (LOS), and costs—as the dependent variable. Studies that recruited less than 75 patients were excluded because the research team assumes that these studies bear the risk to be underpowered.

#### Patients

The included patients were adults (at least 18 years old) with a severe infection. For this review, pneumonia, BSI or bacteraemia, sepsis, severe sepsis, or septic shock were considered severe infections. Studies specifically focused on meningitis, endocarditis or infections in burn and transplant patients were excluded as the literature showed that treatment effects are expected to largely deviate from any common effect.

#### Intervention

The intervention of interest concerned empiric AAT versus IAAT. Empiric antibiotic therapy is defined as all non-definitive therapy and refers to antibiotics given prior to the result of the final culture and the antibiotic sensitivity tests [23]. Studies that did not specify the used definition of AAT or IAAT were excluded. Studies comparing two or more types of antibiotics were excluded.

#### Outcome

Outcomes were assessed in terms of mortality, hospital LOS, and costs.

### Study appraisal

Two reviewers (KM and AL) independently performed the initial scan of titles and abstracts of all retrieved citations by using standardized screening forms. Both reviewers documented the reasons for exclusion. Full-text copies of all potentially relevant studies were obtained and further checked for inclusion. Any discrepancies between reviewers were resolved by discussion. Continuing disagreements were settled by a third reviewer (NC or AV). Additional sources that had been cross-referenced from the Medline search results were included if they met the criteria above. The quality of the articles was evaluated by using the Downs and Black quality assessment method, which is a list of 27 criteria to evaluate both randomized and non-randomized trials [24]. This scale assesses study reporting, external validity, internal validity, and power of non-randomized studies and has been ranked in the top six quality assessment scales suitable for use in systematic reviews [25,26]. As had been done in other reviews using the Downs and Black scale [27-29], the tool was modified slightly for use in this particular review. Specifically, the scoring for question 27 dealing with statistical power was simplified to a choice of awarding either 1 point or 0 points, depending on whether there was sufficient power to detect a clinically important effect. The criterion was that to detect a 10% difference, assuming power of 0.90 and alpha of 0.05. The Downs and Black scores were grouped into the following 4 quality levels: excellent (26 to 28), good (20 to 25), fair (15 to 19) and poor (less than 14) [29]. Only articles with a quality level of good or excellent were retained.

### Data extraction

Data extraction was completed independently by two reviewers (KM and AL), who used a standardized data collection form. The following data were extracted and reported: (1) data on study setting and patient population as possible confounding factors, (2) definition and incidence of the (I)AAT, and (3) definition and measurement of outcome variables (in terms of mortality, hospital LOS, and costs among patients given AAT versus IAAT). In case of disagreement between the two reviewers, a third reviewer (NC or AV) extracted the data.

#### Study characteristics

For every included study, descriptive data on the study setting (that is, study design, geographic location of the study, baseline characteristics, study years, and sample size) and patient characteristics (that is, source of infection and severity scale) were collected.

#### Definition and measuring incidence of (I)AAT

We reviewed how empiric (I)AAT was defined and measured. We assessed which evidence-based elements, such as therapy dose, route, and timing, were evaluated. Empiric antibiotic therapy is defined as all non-definitive therapy and refers to antibiotics given prior to the result of the final culture and the antibiotic sensitivity tests [23].

#### Measurement of the dependent variable

The outcome was measured as mortality, LOS, and costs for patients given empirical (I)AAT. The time span of mortality assessment was also registered.

### Data analysis

Data were analyzed by using R (a language and environment for statistical computing) [30]. All reported *P* values were two-sided; *P* <0.05 was considered to indicate statistical significance. A random-effects meta-analysis using the DerSimonian-Laird estimator obtained risk ratios (RRs) and 95% confidence intervals (CIs) for mortality rate reductions [31]. Heterogeneity of the study results was assessed by using the Cochran Q test and the Higgins I^2^ test. The following thresholds were used to quantify heterogeneity: *P* <0.10 in Cochran’s Q test and I^2^ ≤ 25% for low, 25% < I^2^ < 50% for moderate, and I^2^ ≥ 50% for high. Funnel plots assessed publication bias. Sensitivity analysis identified heterogeneous studies that influenced the meta-analysis. Meta-regression was used to examine the impact of study characteristics on study effect size and heterogeneity.

## Results

### Results of the search

**Figure 1:**
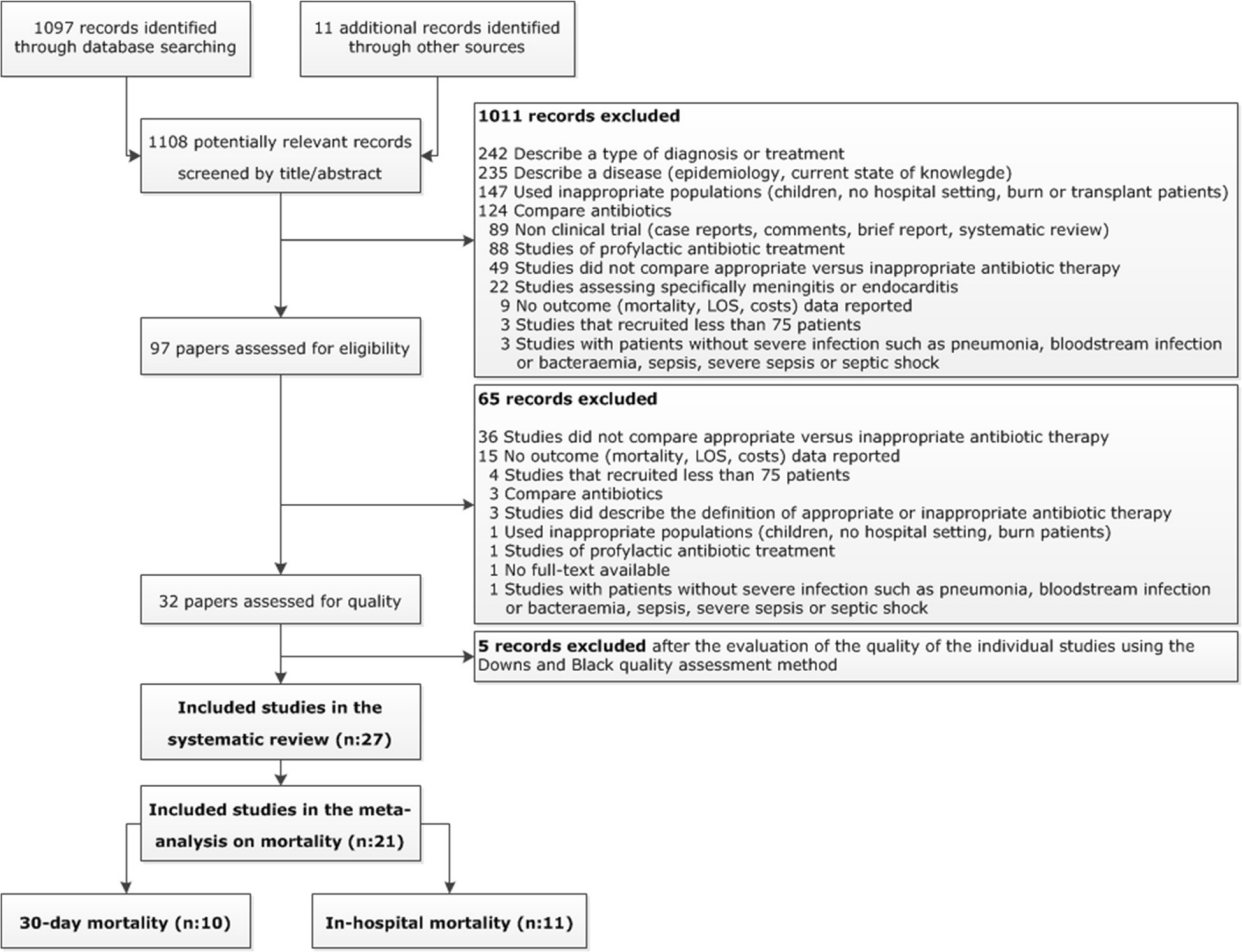
**PRISMA (Preferred Reporting Items of Systematic reviews and Meta-Analyses) diagram for study selection.** LOS, length of stay.

The initial database search identified 1,097 unique citations. Review of the reference lists of included studies identified 11 additional studies. After critical assessment of these 1,108 publications, 32 individual trials [8,12,19,21,22,32-58] fulfilled the inclusion criteria and were considered for further analysis (Figure 1). After quality assessment of the individual studies, 27 studies [8,12,19,21,22,33,34,36-48,50-52,54-57] were included in the systematic review.

### Study characteristics

Characteristics of the 27 included studies are presented in Table 1. The studies were conducted in Asia (n = 9) [8,12,21,41,44,50-52,55], North America (n = 8) [22,33,34,36,37,42,45,54], Europe (n = 6) [19,38,46,48,56,57], and the Middle East (n = 2) [43,47], and two studies were multinational [39,40]. Eight studies (29.6%) were multicenter trials (range 2 to 60) [12,34,38-40,48,50,56]. Twenty studies (74.1%) were conducted in university or teaching hospitals [8,19,21,22,33,36,37,40-42,44-46,48,51,52,54-57], three studies (11.1%) combined university and general hospitals [12,38,39], two studies (7.4%) were performed in general hospitals [47,50], and two studies (7.4%) did not mention the nature of the site [34,43]. Twenty-three studies (85.2%) reported on retrospective analysis [8,12,19,21,22,33,34,37,39-46,48,51,52,54,56,57]. Included studies covered a total of 15,306 patients, with an average of 567 patients per study (range 76 to 5,715). The severe infection was BSI or bacteremia in 15 studies (55.5%) [8,12,19,21,22,33,36,37,39,46,47,50,52,55,56], pneumonia in six studies (22.2%), [34,41,44,48,51,57], and sepsis in three studies [38,42,45]; two studies described severe sepsis or septic shock [40,54]. Severity of illness was reported in 23 studies (85.2%) using a variety of severity indexes, including the Acute Physiology and Chronic Health Evaluation (APACHE) II [59], Charlson index [60], the Sequential Organ Failure Assessment (SOFA) [61], Simplified Acute Physiology Score (SAPS) II [62], Multiple Organ Dysfunction Scale (MODS) [63], Pitt Bacteremia score [64], and McCabe’s classification [65]. A significant difference (*P* = 0.04) in illness severity between the two groups was found in two studies [46,50]. However, nine studies [19,33,38,40,50-52,54,55] did not compare the severity of illness between patients with IAAT versus AAT.

**Table 1:** **Characteristics of 27 included studies in the systematic review**

**Reference**	**Study year(s)**	**Location**	**Design**	**Center**		**Number of patients**	**Outcome**	**Main type of infection**	**Severity index scale and significance difference**
				**Number**	**Type**				
Kim *et al*. [21]^a^	1998-2001	Korea	R	1	U	127	M	MRSA bacteremia	McCabe’s classification, Jackson: NS
Kang *et al*. [12]^a^	1998-2002	Korea	R	2	U, G	286	M	Antibiotic-resistant Gram-negative Bacilli BSI	APACHE II: NS
Micek *et al*. [33]^a^	1997-2002	USA	R	1	U	305	M	*Pseudomonas aeruginosa* BSI	SAPS II: NC
Luna *et al*. [34]	1999-2003	Argentina	P	6	NM	76	M	Pneumonia (VAP)	APACHE II: NS
Kim *et al*. [8]	1998-2001	South Korea	R	1	U	238	M	SAB	McCabe’s classification, Jackson: NS
Scarsi *et al*. [22]^a^	2001-2003	USA	R	1	U	884	M	Gram-negative BSI	Charlson index: NS
Marschall *et al*. [36]^a^	2006-2007	USA	P	1	T	250	M LOS	Gram-negative bacteremia	Charlson index, McCabe’s classification: NS
Shorr *et al*. [37]^a^	2002-2004	USA	R	1	U	291	M LOS C	MRSA infection	NM
Rodríguez-Baño *et al*. [38]^a^	2003	Spain	P	59	U, G	209	M	Sepsis	Charlson index: NC
Ammerlaan *et al*. [39]^a^	2007	West European countries	R	60	T, G	334	M	SAB	Modified Charlson index: NS
Erbay *et al*. [19]^a^	2005-2008	Turkey	R	1	U	103	M	Acinetobacter baumannii bacteremia	APACHE II: NC
Kumar *et al*. [40]^a^	1996-2005	Canada, USA, Saudi Arabia	R	22	U	5,715	M	Septic shock	APACHE II: NC
Tseng *et al*. [41]^a^	2005-2007	Taiwan	R	1	T	163	M	Pneumonia	Charlson index: NC
Micek *et al*. [42]^a^	2002-2007	USA	R	1	U	760	M	Gram-negative sepsis	APACHE II, Charlson index: NS
Paul *et al*. [43]^a^	1999-2007	Israel	R	1	NM	510	M	MRSA bacteremia	NM
Joung *et al*. [44]^a^	2000-2006	Korea	R	1	U	116	M	Pneumonia (HAP) Acinetobacter baumannii	APACHE II: NS
Shorr *et al*. [45]	2002-2007	USA	R	1	U	760	LOS	Gram-negative sepsis	APACHE II, Charlson index: NS
Suppli *et al*. [46]^a^	2002-2005	Denmark	R	1	T	196	M	Enterococcal BSI	Charlson index: NS, except score 0 (*P* = 0.04)
Reisfeld *et al*. [47]^a^	2005-2007	Israel	R	1	G	378	M	Gram-negative bacteremia	NM
Wilke *et al*. [48]^a^	2007	Germany	R	5	T	221	M LOS C	Pneumonia (VAP, HAP)	NM
Lye *et al*. [50]^a^	2007-2009	Singapore	R	2	G	675	M	Gram-negative bacteremia	APACHE II <0.001; Charlson index: NS
Tseng *et al*. [51]	2007-2008	Taiwan	R	1	U	163	M	Pneumonia (VAP)	APACHE II, Charlson index, SOFA: NC
Chen *et al*. [52]	2006-2011	China	R	1	T	118	M	SAB	APACHE II: NC
Labelle *et al*. [54]^a^	2002-2007	USA	R	1	T	436	M	Septic shock	APACHE II, Charlson index: NC
Chen *et al*. [55]	2008-2009	Taiwan	P	1	U	937	M, LOS	BSI	MEDS, Charlson index: NC
Frakking *et al*. [56]^a^	2008-2010	The Netherlands	R	8	U	232	M	ESBL bacteremia	Pitt bacteremia score: NS
Tumbarello *et al*. [57]^a^	2008-2010	Italy	R	1	U	110	M	*Pseudomonas aeruginosa* pneumonia	SAPS II, SOFA: NS

^a^Twenty-one included studies in meta-analysis. APACHE II, Acute Physiology and Chronic Health Evaluation II; BSI, bloodstream infection; C, costs; ESBL, extended-spectrum β-lactamase; G, general hospital; HAP, hospital-acquired pneumonia; LOS: Length Of Stay; M, mortality; MEDS, Mortality in Emergency Department Sepsis; MRSA, Methicillin-resistant *Staphylococcus Aureus*; NC, no comparison; NM, not mentioned; NS, not significant; P, prospective; R, retrospective; SAB, *Staphylococcus Aureus* bacteraemia; SAPS II, Simplified Acute Physiology Score II; SOFA, Sequential Organ Failure Assessment; T, teaching hospital, U, university hospital; USA, United States of America; VAP, ventilator-associated pneumonia.

### Data on definition and measurement of (I)AAT

Data on the definition and the incidence of (I)AAT were presented in Table 2. A spectrum of definitions exists in the literature concerned. Fifteen (55.6%) studies included a definition of AAT, four studies (14.8%) mentioned a definition of IAAT, and eight studies (29.6%) defined both. Thirty-two (94.1%) of the 34 definitions mentioned the element ‘matching with the *in vitro* susceptibility’ or ‘intermediate or full *in vitro* resistance’. Other frequently mentioned definitions items were the timing of administration (n = 24, 70.6%), the correct dose (n = 8, 23.5%), and the correct indication for the antibiotics (n = 6, 17.6%).

**Table 2:** **Definition and incidence of (in)appropriate antibiotic therapy in the reviewed studies**

**Reference**	**Appropriate empiric antibiotic therapy**											**Inappropriate empiric antibiotic therapy**							
	**Aspects of appropriate antibiotic therapy**											**Aspects of inappropriate antibiotic therapy**							
	**Definition**	**According to the culture**	**Timing**	**Dose**	**According to guidelines**	**Route**	**Indication**	**Duration**	**No known contraindication**	**Frequency**	**Number of items**	**Definition**	**Intermediate or full** ***in vitro*** **resistance**	**Timing**	**Omission**	**Indication**	**Route**	**Number of items**	**% IAAT**
Kim *et al*. [21]^a^	**Y**	Y	Y	N	N	Y	N	N	N	N	**3**	**N**							76.38
Kang *et al*. [12]^a^	**N**											**Y**	Y	Y	Y	N	N	**3**	52.80
Micek *et al*. [33]^a^	**N**											**Y**	Y	N	Y	N	N	**2**	24.59
Luna *et al*. [34]	**Y**	Y	N	N	Y	N	N	N	N	N	**2**	**Y**	Y	Y	N	N	N	**2**	68.42
Kim *et al*. [8]	**Y**	Y	Y	N	N	Y	N	N	N	N	**3**	**N**							49.16
Scarsi *et al*. [22]^a^	**Y**	Y	Y	Y	Y	N	N	N	N	N	**4**	**Y**	Y	Y	N	N	N	**2**	14.14
Marschall *et al*. [36]^a^	**Y**	Y	Y	N	N	N	N	N	N	N	**2**	**Y**	Y	N	Y	N	N	**2**	31.6
Shorr *et al*. [37]^a^	**Y**	Y	Y	N	N	N	N	N	N	N	**2**	**N**							76.98
Rodriguez-Bano *et al*. [38]^a^	**Y**	Y	Y	Y	N	Y	N	N	N	N	**4**	**N**							78.95
Ammerlaan *et al*. [39]^a^	**Y**	Y	Y	N	N	Y	N	N	N	N	**3**	**Y**	Y	Y	Y	Y	Y	**5**	28.14
Erbay *et al*. [19]^a^	**Y**	Y	Y	Y	Y	Y	N	N	N	N	**5**	**N**							58.25
Kumar *et al*. [40]^a^	**Y**	Y	Y	N	N	N	N	N	N	N	**2**	**Y**	Y	Y	N	N	N	**2**	19.88
Tseng *et al*. [41]^a^	**Y**	Y	N	N	N	N	Y	N	N	N	**2**	**Y**	Y	N	N	Y	N	**2**	49.26
Micek *et al*. [42]^a^	**Y**	Y	Y	N	N	N	Y	Y	N	N	**4**	**N**							31.32
Paul *et al*. [43]^a^	**Y**	Y	Y	N	N	N	N	N	N	N	**2**	**N**							67.06
Joung *et al*. [44]^a^	**Y**	Y	Y	Y	N	Y	N	N	N	N	**4**	**Y**	Y	Y	N	N	N	**2**	57.76
Shorr *et al*. [45]	**N**											**Y**	Y	Y	Y	N	N	**3**	31.30
Suppli *et al*. [46]^a^	**Y**	Y	Y	Y	Y	N	Y	Y	Y	N	**7**	**N**							25.51
Reisfeld *et al*. [47]^a^	**Y**	Y	N	Y	N	N	N	N	N	Y	**3**	**N**							39.95
Wilke *et al*. [48]^a^	**Y**	N	N	N	Y	N	N	N	N	N	**1**	**N**							51.58
Lye *et al*. [50]^a^	**Y**	Y	N	Y	Y	N	N	N	N	N	**3**	**N**							43.56
Tseng *et al*. [51]	**Y**	N	N	N	N	N	Y	N	N	N	**1**	**N**							56.44
Chen *et al*. [52]	**Y**	Y	Y	N	N	N	N	N	N	N	**2**	**N**							38.98
Labelle *et al*. [54]^a^	**Y**	Y	Y	N	N	N	N	N	N	N	**2**	**N**							51.88
Chen *et al*. [55]	**Y**	Y	Y	Y	Y	Y	N	N	N	N	**5**	**N**							27.21
Frakking *et al*. [56]^a^	**Y**	Y	Y	N	N	N	N	Y	N	N	**3**	**N**							63.36
Tumbarello *et al*. [57]^a^	**N**											**Y**	Y	N	N	N	N	**1**	50.91
Total	**23**	**21**	**17**	**8**	**7**	**7**	**4**	**3**	**1**	**1**		**11**	**11**	**7**	**5**	**2**	**1**		

^a^Included in the meta-analysis. IAAT, inappropriate antibiotic therapy; N, no; Y, yes.

The percentage of empiric IAAT showed an enormous range from 14.1% to 78.9% (median of 49, 26%, interquartile range 28.1% to 57.8%). The magnitude of this range can be explained in part by the differences in the definitions, settings, diseases, and infectious agents. Because of this considerable heterogeneity, it may be misleading to quote an average value for the incidence. However, 13 (48.1%) of these 27 studies described an incidence of IAAT of 50% or more.

### Measurement of the dependent variable

Outcome was measured as mortality, LOS, and costs. A meta-analysis was conducted to quantify the effect of appropriateness in empiric antibiotics on mortality. The number of studies that assess the total LOS [48,55], LOS after infection onset [36,45], and the costs [37,48] were very small. Therefore, these results are presented in a descriptive manner only.

#### Mortality

**Figure 2:**
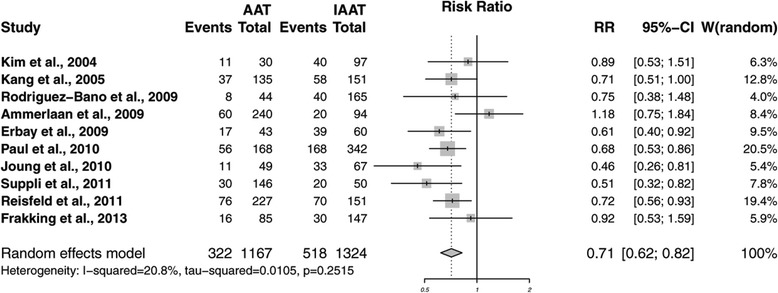
**Forest plot showing the effectiveness of appropriateness empirical antibiotics in severe infections on 30-day mortality.**

**Figure 3:**
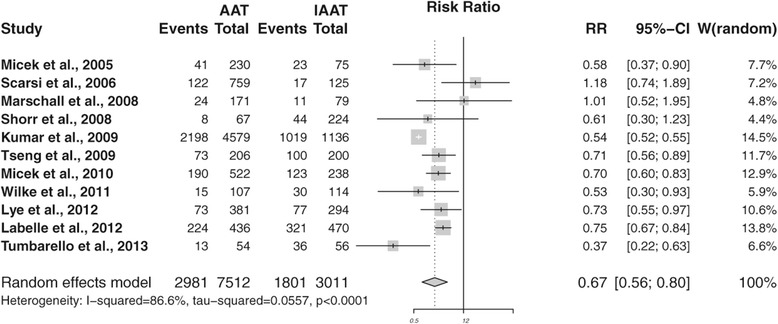
**Forest plot showing the effectiveness of appropriateness empirical antibiotics in severe infections on in-hospital mortality.**

In total, 26 studies [8,12,19,21,22,33,34,36-44,46-48,50,51,54-57] reported mortality as an outcome variable in patients with severe infection treated with (I)AAT. However, the time span of mortality assessment varied from 28 [34,55] to 30 [12,19,21,38,39,43,44,46,47,56] to 60 [51] days to 12 weeks [8]. Eleven studies [22,33,36,37,40-42,48,50,54,57] assessed in-hospital mortality. Given methodological considerations, meta-analysis on the effect of AAT on 30-day mortality (n = 10) and in-hospital mortality (n = 11) was conducted separately (Table 3). Five [12,19,43,44,46] of the 10 studies reporting on 30-day mortality showed a significant lower mortality for patients treated with AAT compared with those treated with IAAT. Meta-analysis for 30-day mortality revealed an RR of 0.71 (95% CI 0.62 to 0.82; *P* <0.0001) in favor of AAT, without significant heterogeneity: Cochran’s Q = 11.37, 9 degrees of freedom (d.f.), *P* = 0.252; I^2^ = 20.8 (0% to 61%) (Figure 2). Of the 11 trials [22,33,36,37,40-42,48,50,54,57] included in the meta-analysis on in-hospital mortality, eight trials [33,40-42,48,50,54,57] yielded significant lower mortality ratios in patients receiving AAT. Meta-analysis for in-hospital mortality revealed that an RR of 0.67 (95% CI 0.56 to 0.80; *P* <0.0001) in favor of AAT. However, there was significant heterogeneity: Cochran’s Q = 74.45, 10 d.f., *P* <0.0001; I^2^ = 86.6 (77.8% to 91.9%) (Figure 3). Funnel plots displayed an asymmetrical pattern for in-hospital mortality but not for 30-day mortality studies. The results of the sensitivity analysis suggest that three studies contribute to residual heterogeneity; removing them from the meta-analysis would reduce variability between studies. However, because this did not affect the results, these studies were retained. Meta-regression revealed that study quality (Down and Black score) (*P* = 0.003), inclusion of a definition of appropriate antibiotic usage (*P* = 0.0194), and studies reporting outcome for sepsis (*P* = 0.0001) significantly influenced the meta-analysis on in-hospital mortality.

**Table 3:** **Summary of mortality data included in the meta-analysis**

**Reference**	**Time of mortality assessment**	**AAT mortality rate, %**	**IAAT mortality rate, %**	***P*** **value**
Kim *et al*. [21]	30	36.67	41.24	0.36
Kang *et al*. [12]	30	27.41	38.41	0.049
Micek *et al*. [33]	IHM	17.83	30.67	0.018
Scarsi *et al*. [22]	IHM	16.07	13.60	0.48
Marschall *et al*. [36]	IHM	14.03	13.92	1.0
Shorr *et al*. [37]	IHM	11.94	19.64	0.15
Rodríguez-Baño *et al*. [38]	30	18.18	24.24	0.3
Ammerlaan *et al*. [39]	30	25.00	21.27	NS
Erbay *et al*. [19]	30	39.53	65.00	0.011
Kumar *et al*. [40]	IHM	48.00	89.70	<0.0001
Tseng *et al*. [41]	IHM	35.44	50.00	OR 2.17 (1.4-3.38) 0.001
Micek *et al*. [42]	IHM	36.40	51.68	<0.001
Paul *et al*. [43]	30	33.33	49.12	0.001
Joung *et al*. [44]	30	22.45	49.25	<0.0001
Suppli *et al*. [46]	30	20.55	40.00	0.009
Reisfeld *et al*. [47]	30	33.48	46.36	OR 1.4 (0.86-2.29) (NS)
Wilke *et al*. [48]	IHM	14.02	26.32	0.021
Lye *et al*. [50]	IHM	19.16	26.19	OR 0.67 (0.46-0.96) 0.03
Labelle *et al*. [54]	IHM	51.38	68.30	<0.001
Frakking *et al*. [56]	30	18.82	20.41	NS
Tumbarello *et al*. [57]	IHM	24.07	64.29	<0.001

AAT, appropriate antibiotic therapy; IAAT, inappropriate antibiotic therapy; IHM, in-hospital mortality; OR, odds ratio; NS, not significant.

The studies on 28-day [34,55] and 60-day [51] mortality reported significantly higher mortality ratios in patients receiving IAAT: respectively *P =* 0.007 [34], *P =* 0.001 [55], and *P =* 0.023 [51]. The one study [8] that measures the mortality rate at 12 weeks did not reveal a significant difference (Table 4).

**Table 4:** **Overview of studies evaluating the mortality rate at 28 and 60 days and 12 weeks**

**Reference**	**Time of mortality assessment**	**AAT mortality rate**	**IAAT mortality rate**	**Significant differences**
Luna *et al*., 2006 [34]	28 days	29.17	63.46	0.007
Chen *et al*., 2013 [55]	28 days	9.09	38.04	0.001
Tseng *et al*., 2012 [51]	60 days	28.17	55.43	0.023
Kim *et al*., 2006 [8]	12 weeks	28.10	38.46	NS

AAT, appropriate antibiotic therapy; IAAT, inappropriate antibiotic therapy; NS, not significant.

#### LOS and costs

Four studies reported the effect on LOS: total LOS [48,55] or LOS after the onset of infection [36,45]. In one of the two studies [45], the mean LOS after infection onset was significantly (*P* = 0.022) higher in the group sepsis patients with IAAT. This indicates that IAAT independently increased the median attributable LOS by 2 days. However, the study by Marschall *et al*. [36] found no significant differences in LOS post-onset (*P* = 0.09) in patients with Gram-negative bacteraemia. Appropriately treated patients with ventilator-associated pneumonia had a significantly shorter total LOS (*P* = 0.022) [48]. Nevertheless, Chen *et al*. [55] found no differences in the total LOS of patients with community-onset bloodstream infections. The costs were assessed in only two studies [37,48]. The total costs for patients with IAAT were significantly higher in both studies (*P* ≤0.01).

## Discussion

The incidence of patients with severe infections is substantial. Previous studies confirmed—as proven by the low number needed to treat—that correct antibiotic treatment is a crucial determinant of therapeutic success [66]. Therefore, a systematic review with meta-analysis was conducted to investigate the incidence and consequences of IAAT on the outcome in hospitalized patients with infection.

Definitions and criteria items used to denote (I)AAT varied substantially between studies. However, most definitions included the criterion ‘matching with the *in vitro* susceptibility’ or ‘intermediate or full *in vitro* resistance’. The timing of administration of the antibiotics was taken into account in only 71% of the definitions. Timing of admission is, however, an important aspect of adequate antibiotic therapy. In patients with septic shock, each hour of delay in antimicrobial therapy is associated with an average decrease in survival of 7.6% [13]. Rivers *et al*. [67] showed that early goal-directed therapy provides significant benefits with respect to outcome in patients with severe sepsis and septic shock. For patients with *Staphylococcus aureus* bacteraemia, the breakpoint between delayed and early treatment was 44.75 hours, and delayed treatment was found to be an independent predictor of infection-related mortality [68]. Based on this heterogeneity in the definitions, it was impossible to estimate the overall incidence of IAAT. However, IAAT ranged from 14.1% to 78.9%, and 46.4% of studies described an incidence of IAAT of 50% or more. Given this high incidence, health-care professionals must become aware of this problem. Moreover, in an era of rising antimicrobial resistance rates, choosing empiric AAT is an increasing challenge. The meta-analysis, involving 13,014 patients, suggests that the empiric AAT reduces 30-day mortality (RR 0.71, 95% CI 0.62 to 0.82) and in-hospital mortality (RR 0.67, 95% CI 0.56 to 0.80). In addition, empiric AAT positively affects LOS and costs.

Strengths of this study include the comprehensive search strategy, the methodological quality assessment, and the random-effects model analysis combined with meta-regression. Besides the methodological strengths, the study has limitations. First, the present findings should be interpreted in the context of the included studies and their limitations: the heterogeneity in patients’ characteristics, definitions of IAAT, and the time span of outcome assessment. Second, the lack of RCTs is this review could be seen as a major limitation. The lack of RCTs regarding this topic stems from obvious ethical constraints. Given the methodological heterogeneity of the included (retro- and prospective) observational studies, an overall meta-analysis was impossible. Meta-analysis was performed for 30-day and in-hospital mortality only. Third, several potential biasing and confounding elements might have hampered this meta-analysis. The reported diseases and the diagnosis process, the study quality quantified by the Downs and Black instrument, the quality of the health-care systems in the different countries, and the definitions of adequate antibiotic therapy had a marked influence on the meta-analysis of in-hospital mortality. Nevertheless, we aggregated all reported diseases to avoid a small numbers problem. Probably the cleanest data for assessing the impact of (I)AAT would be for bacteremia, as this is the infection that can most accurately be defined. Fourth, this analysis does not cover all areas, such as fungemiae. However, this limitation creates opportunities for further research. Fifthly, we used the (criteria of the) definitions used in the included studies. Most of the studies approached the definition one-sided and used only the criteria ‘matching with the culture’ and ‘according to the guidelines’. However, appropriateness of antibiotic treatment is related not only to the substance itself but also to dosing or administration route (or both) of the antibiotic. Finally, during this review, we focused on (in)appropriate antibiotic therapy. Off course, inappropriate therapy is not only determined by the antibiotic used. Further research could focus on other aspects of (in)appropriate therapy.

## Conclusions

This systematic review demonstrates a very high incidence of IAAT in patients with severe bacterial infection, such as BSI, pneumonia, sepsis, or septic shock. Accurate empirical treatment of these severe infections is not a simple process seen in currently reported rates of IAAT. Meta-analysis provides evidence that empiric inappropriate use of empiric antibiotics increases 30-day and in-hospital mortality in these patients. Clinicians should be aware of this problem, and further improvement actions should be taken. Inappropriate antibiotic treatment stems from several causes, mainly due to resistance; therefore, it is not easy to find the most appropriate treatment option. As long as general recommendations about antibiotic stewardship are missing, problems will remain. Computerized decision support, including complex and locally calibrated decision algorithms [69,70] or early molecular identification or both, might be helpful.

## Key messages

- The definitions of IAAT varied. Nevertheless, almost every definition included the element ‘matching with the *in vitro* susceptibility’ or ‘intermediate or full *in vitro* resistance’.
- This systematic review demonstrates a very high incidence of empiric IAAT in patients with severe infection, such as BSI, pneumonia, sepsis, or septic shock.
- Meta-analysis provides evidence that empiric IAAT increases 30-day and in-hospital mortality in patients with a severe infection.
- Clinicians should be aware of this problem, and further improvement actions should be taken. Further computerized decision support needs to be developed.

## Additional file

Additional file 1:
**Appendix 1.** Literature search strategy. MeSH, Medical Subject Headings. Appendix 2. Description of exclusion criteria. Appendix 3. Downs and Black checklist for methodological quality assessment of included studies. Appendix 4. Data collection tool. Appendix 5. Reference of studies included in systematic review but not in the meta-analysis.

## Abbreviations

AAT: appropriate antibiotic therapy
BSI: bloodstream infection
CI: confidence interval
d.f.: degrees of freedom
IAAT: inappropriate antibiotic therapy
LOS: length of stay
RCT: randomized controlled trial
RR: risk ratio
