# Supplementary material for: Incidence and outcome of inappropriate in-hospital empiric antibiotics for severe infection: a systematic review and meta-analysis
Source: Crit Care. 2015 Feb 16;19(1):63. doi: 10.1186/s13054-015-0795-y (PMC4358713; doi:10.1186/s13054-015-0795-y)
Supplement: Additional file 1: — Appendix 1. Literature search strategy. MeSH, Medical Subject Headings. Appendix 2. Description of exclusion criteria. Appendix 3. Downs and Black checklist for methodological quality assessment of included studies. Appendix 4. Data collection tool. Appendix 5. Reference of studies included in systematic review but not in the meta-analysis. [file 13054_2015_795_MOESM1_ESM.docx]

**Additional file 1: digital content**

- Appendix 1 Literature search strategy
- Appendix 2 Description of exclusion criteria
- Appendix 3 Downs and Black checklist for methodological quality assessment of included studies
- Appendix 4 Data collection tool
- Appendix 5 Reference of studies only included in systematic review but not in the meta-analysis

**Appendix 1. Literature search strategy**

| **Used query** |
| --- |
| "antibiotic"[All Fields] AND "infection"[All Fields] AND ("appropriate"[All Fields] OR "inappropriate"[All Fields] OR "adequate"[All Fields] OR "inadequate"[All Fields]) AND ("outcome"[All Fields] OR "mortality"[All Fields] OR "survival rate"[All Fields] OR "cost"[All Fields] OR "length of stay"[All Fields]) AND "2004/08/20"[PDAT] : "2014/08/20"[PDAT] AND "humans"[MeSH Terms] AND ("loattrfull text"[sb] AND (English[lang] OR French[lang] OR Dutch[lang] OR German[lang])) |
| **Result: 1097** |

**Appendix 2. Description of exclusion criteria**

| **Reason for exclusion** | **Description** |
| --- | --- |
| Describe a type of diagnosis or treatment | Article describe a type of diagnosis or treatment not related to appropriate antibiotics |
| Describe a disease | Article describe epidemiology, current state of knowledge |
| Using inappropriate populations | Studies in pediatrics, burn or transplant patients, no hospital setting, veteran affairs center |
| Compare or describe antibiotics | Studies comparing the effectiveness of two or more antibiotics |
| Non clinical trials | Papers were guidelines, editorials, systematic reviews, case reports, letters, or comments |
| Study of prophylactic antibiotic treatment | |
| No comparison between inappropriate and appropriate antibiotic therapy | |
| Studies assessing specifically meningitis and endocarditis, where treatment effects are expected to largely deviate from any common effect | |
| No (quantitative) outcome | Studies report no (quantitative) data on mortality, length of stay of costs |
| Studies that recruited less than 75 patients | |
| Other infection than pneumonia, bloodstream infection (BSI) or bacteremia, sepsis, severe sepsis or septic shock | |
| Study compared survivors versus non-survivors | Studies compared survivors and non-survivors, without report not about inappropriate antibiotics |
| No full-text available | |
| The study give no definition for appropriate or inappropriate antibiotic therapy | |

**Appendix 3. Downs and Black checklist for methodological quality assessment of included studies**

**Appendix 3.1 Criteria per Downs and Black item**

1. Is the hypothesis/aim/objective of the study clearly described?
2. Are the main outcomes to be measured clearly described in the Introduction or Methods section?
3. Are the characteristics of the patients included in the study clearly described?
4. Are the interventions of interest clearly described?
5. Are the distributions of principal confounders in each group of subjects to be compared clearly described?
6. Are the main findings of the study clearly described?
7. Does the study provide estimates of the random variability in the data for the main outcomes?
8. Have all important adverse events that may be a consequence of the intervention been reported?
9. Have the characteristics of patients lost to follow-up been described? (No patients lost to follow-up = 1 point)
10. Have actual probability values been reported (e.g. 0.035 rather than <0.05) for the main outcomes except where the probability value is less than 0.001?
11. Were the subjects asked to participate in the study representative of the entire population from which they were recruited?
12. Were the patients who were prepared to participate representative of the entire population from which they were recruited?
13. Were the staff, places, and facilities where the patients were treated, representative of the treatment the majority of patients receive?
14. Was an attempt made to blind study subjects to the intervention they have received?
15. Was an attempt made to blind those measuring the main outcomes of the intervention?
16. If any of the results of the study were based on “data dredging”, was this made clear?
17. In trials and cohort studies, do the analyses adjust for different lengths of follow-up of patients, or in case-control studies, is the time period between the  intervention and outcome the same for cases and controls? (survival analysis should be answer by yes)
18. Were the statistical tests used to assess the main outcomes appropriate?
19. Was compliance with the intervention/s reliable?
20. Were the main outcome measures used accurate (valid and reliable)? (always 1 point because definitions were reported: inclusion criteria)
21. Were the patients in different intervention groups (trials and cohort studies) or were the cases and controls (case-control studies) recruited from the same population? (always 1 point because patients are recruited from the same study population)
22. Were study subjects in different intervention groups (trials and cohort studies) or were the cases and controls (case-control studies) recruited over the same period of time? (always 1 point because patients are recruited from the same study population during the same time)
23. Were study subjects randomized to intervention groups? (always 0 because all non-randomized studies should be answered no)
24. Was the randomized intervention assignment concealed from both patients and health care staff until recruitment was complete and irrevocable? (always 0 because all non-randomized studies should be answered no)
25. Was there adequate adjustment for confounding in the analyses from which the main findings were drawn? (1 point if a difference in patient characteristics or severity index was corrected for)
26. Were losses of patients to follow-up taken into account?
27. Did the study have sufficient power to detect a clinically important effect?

Downs and Black score ranges were grouped into the following 4 quality levels:

- excellent (26 to 28),
- good (20 to 25),
- fair (15 to 19) and
- poor (less than 14)(1).

**Appendix 3.2 Results of the Downs and Black checklist per study**

| **First author / year** | **Questions of the Downs and Black tool** | | | | | | | | | | | | | | | | | | | | | | | | | | | | | | | | |  |  |
| --- | --- | --- | --- | --- | --- | --- | --- | --- | --- | --- | --- | --- | --- | --- | --- | --- | --- | --- | --- | --- | --- | --- | --- | --- | --- | --- | --- | --- | --- | --- | --- | --- | --- | --- | --- |
|  | **Reporting** | | | | | | | | | | **External validity** | | | | **Internal validity** | | | | | | | | **Internal validity - confounding** | | | | | | | **Power** | | **Total score** | | | |
|  | **1** | **2** | **3** | **4** | **5** | **6** | **7** | **8** | **9** | **10** | | **11** | **12** | **13** | | **14** | **15** | **16** | **17** | **18** | **19** | **20** | | **21** | **22** | **23** | **24** | **25** | **26** | | **27** |  |  |  |  |
| Kim et al., 2004 (2) | **1** | **1** | **1** | **1** | **1** | **1** | **1** | **1** | **0** | **1** | | **1** | **1** | **1** | | **1** | **0** | **1** | **1** | **1** | **1** | **1** | | **1** | **1** | **0** | **0** | **1** | **1** | | **0** | **22** | **good** | |  |
| Kang et al., 2005 (3) | **1** | **1** | **1** | **1** | **1** | **1** | **1** | **0** | **1** | **1** | | **1** | **1** | **1** | | **1** | **0** | **0** | **1** | **1** | **1** | **1** | | **1** | **1** | **0** | **0** | **1** | **1** | | **1** | **22** | **good** | |  |
| Mueller et al., 2005 (4) | **1** | **0** | **1** | **1** | **1** | **1** | **1** | **0** | **1** | **0** | | **1** | **1** | **1** | | **1** | **0** | **1** | **1** | **1** | **1** | **1** | | **1** | **1** | **0** | **0** | **0** | **1** | | **0** | **19** | **far** | |  |
| Micek et al., 2005 (5) | **1** | **1** | **1** | **1** | **0** | **1** | **1** | **0** | **1** | **1** | | **1** | **1** | **1** | | **1** | **0** | **1** | **1** | **1** | **1** | **1** | | **1** | **1** | **0** | **0** | **1** | **1** | | **0** | **21** | **good** | |  |
| Luna et al., 2006 (6) | **1** | **1** | **1** | **1** | **1** | **1** | **1** | **1** | **1** | **1** | | **1** | **0** | **0** | | **1** | **1** | **1** | **1** | **1** | **1** | **1** | | **1** | **1** | **0** | **0** | **1** | **1** | | **0** | **22** | **good** | |  |
| Kim et al., 2006 (7) | **1** | **1** | **1** | **1** | **1** | **1** | **1** | **0** | **1** | **1** | | **1** | **1** | **1** | | **1** | **0** | **1** | **1** | **1** | **1** | **1** | | **1** | **1** | **0** | **0** | **1** | **1** | | **1** | **23** | **good** | |  |
| Scarsi et al., 2006 (8) | **1** | **0** | **0** | **1** | **1** | **1** | **1** | **1** | **1** | **1** | | **1** | **1** | **1** | | **1** | **0** | **1** | **1** | **1** | **1** | **1** | | **1** | **1** | **0** | **0** | **1** | **1** | | **1** | **22** | **good** | |  |
| Fujita et al., 2008(9) | **1** | **0** | **1** | **1** | **0** | **0** | **1** | **0** | **0** | **1** | | **0** | **0** | **0** | | **1** | **0** | **1** | **1** | **1** | **1** | **1** | | **1** | **1** | **0** | **0** | **0** | **1** | | **0** | **14** | **poor** | |  |
| Marschall et al., 2008 (10) | **1** | **1** | **1** | **1** | **1** | **1** | **1** | **1** | **1** | **1** | | **1** | **1** | **1** | | **1** | **1** | **1** | **1** | **1** | **1** | **1** | | **1** | **1** | **0** | **0** | **1** | **1** | | **0** | **24** | **good** | |  |
| Shorr et al., 2008 (11) | **1** | **1** | **1** | **1** | **1** | **1** | **1** | **0** | **0** | **1** | | **1** | **1** | **1** | | **1** | **0** | **1** | **1** | **1** | **1** | **1** | | **1** | **1** | **0** | **0** | **0** | **1** | | **0** | **20** | **good** | |  |
| Rodriguez-Bano et al., 2009 (12) | **1** | **1** | **1** | **1** | **0** | **1** | **1** | **1** | **1** | **1** | | **1** | **1** | **1** | | **1** | **1** | **1** | **1** | **1** | **1** | **1** | | **1** | **1** | **0** | **0** | **0** | **1** | | **0** | **22** | **good** | |  |
| Ammerlaan et al., 2009 (13) | **1** | **1** | **1** | **1** | **1** | **1** | **1** | **0** | **1** | **1** | | **1** | **1** | **1** | | **1** | **0** | **1** | **1** | **1** | **1** | **1** | | **1** | **1** | **0** | **0** | **1** | **1** | | **0** | **22** | **good** | |  |
| Erbay et al., 2009 (14) | **1** | **1** | **1** | **1** | **0** | **1** | **1** | **1** | **0** | **1** | | **1** | **1** | **1** | | **1** | **0** | **1** | **1** | **1** | **1** | **1** | | **1** | **1** | **0** | **0** | **0** | **1** | | **0** | **20** | **good** | |  |
| Kumar et al., 2009 (15) | **1** | **0** | **1** | **1** | **0** | **1** | **1** | **0** | **1** | **1** | | **1** | **1** | **1** | | **1** | **0** | **1** | **1** | **1** | **1** | **1** | | **1** | **1** | **0** | **0** | **0** | **1** | | **1** | **20** | **good** | |  |
| Tseng et al., 2009 (16) | **1** | **1** | **1** | **1** | **0** | **1** | **1** | **0** | **1** | **1** | | **1** | **1** | **1** | | **1** | **0** | **1** | **1** | **1** | **1** | **1** | | **1** | **1** | **0** | **0** | **0** | **1** | | **1** | **21** | **good** | |  |
| Micek et al., 2010 (17) | **1** | **1** | **1** | **1** | **1** | **1** | **1** | **0** | **1** | **1** | | **1** | **1** | **1** | | **1** | **0** | **1** | **1** | **1** | **1** | **1** | | **1** | **1** | **0** | **0** | **1** | **1** | | **1** | **23** | **good** | |  |
| Paul et al., 2010 (18) | **1** | **1** | **1** | **1** | **0** | **1** | **1** | **0** | **1** | **1** | | **1** | **1** | **1** | | **1** | **0** | **1** | **1** | **1** | **1** | **1** | | **1** | **1** | **0** | **0** | **0** | **1** | | **1** | **21** | **good** | |  |
| Joung et al., 2010 (19) | **1** | **1** | **1** | **1** | **1** | **0** | **1** | **0** | **1** | **1** | | **1** | **1** | **1** | | **1** | **0** | **1** | **1** | **1** | **1** | **1** | | **1** | **1** | **0** | **0** | **1** | **1** | | **0** | **21** | **good** | |  |
| Shorr et al., 2011 (20) | **1** | **1** | **1** | **1** | **1** | **1** | **1** | **0** | **0** | **1** | | **1** | **1** | **1** | | **1** | **0** | **1** | **1** | **1** | **1** | **1** | | **1** | **1** | **0** | **0** | **1** | **1** | | **1** | **22** | **good** | |  |
| Suppli et al., 2011 (21) | **1** | **1** | **1** | **1** | **1** | **1** | **1** | **0** | **1** | **1** | | **1** | **1** | **1** | | **1** | **0** | **1** | **1** | **1** | **1** | **1** | | **1** | **1** | **0** | **0** | **1** | **1** | | **0** | **22** | **good** | |  |
| Reisfeld et al., 2011 (22) | **1** | **1** | **1** | **1** | **0** | **1** | **1** | **0** | **1** | **1** | | **1** | **1** | **1** | | **1** | **0** | **1** | **1** | **1** | **1** | **1** | | **1** | **1** | **0** | **0** | **0** | **1** | | **1** | **21** | **good** | |  |
| Wilke et al., 2011 (23) | **1** | **1** | **1** | **1** | **0** | **1** | **1** | **0** | **1** | **1** | | **1** | **1** | **1** | | **1** | **0** | **1** | **1** | **1** | **1** | **1** | | **1** | **1** | **0** | **0** | **0** | **1** | | **1** | **21** | **good** | |  |
| De Rosa et al., 2011 (24) | **1** | **1** | **1** | **1** | **0** | **1** | **1** | **0** | **0** | **1** | | **1** | **1** | **1** | | **1** | **0** | **1** | **1** | **1** | **1** | **1** | | **1** | **1** | **0** | **0** | **0** | **0** | | **0** | **18** | **far** | |  |
| Lye et al., 2012 (25) | **1** | **1** | **1** | **1** | **0** | **1** | **1** | **1** | **1** | **1** | | **1** | **1** | **1** | | **1** | **0** | **1** | **1** | **1** | **1** | **1** | | **1** | **1** | **0** | **0** | **0** | **0** | | **1** | **21** | **good** | |  |
| Tseng et al., 2012 (26) | **1** | **1** | **1** | **1** | **0** | **1** | **1** | **0** | **1** | **1** | | **1** | **1** | **1** | | **1** | **0** | **1** | **1** | **1** | **1** | **1** | | **1** | **1** | **0** | **0** | **0** | **1** | | **0** | **20** | **good** | |  |
| Chen et al., 2012 (27) | **1** | **1** | **1** | **1** | **0** | **1** | **1** | **0** | **1** | **1** | | **1** | **1** | **1** | | **1** | **0** | **1** | **1** | **1** | **1** | **1** | | **1** | **1** | **0** | **0** | **0** | **1** | | **0** | **20** | **good** | |  |
| Kim et al., 2012 (28) | **1** | **1** | **1** | **1** | **0** | **1** | **1** | **0** | **1** | **0** | | **1** | **1** | **1** | | **1** | **0** | **1** | **1** | **1** | **1** | **1** | | **1** | **1** | **0** | **0** | **0** | **1** | | **0** | **19** | **far** | |  |
| Labelle et al., 2012 (29) | **1** | **1** | **1** | **1** | **0** | **1** | **1** | **0** | **1** | **1** | | **1** | **1** | **1** | | **1** | **0** | **1** | **1** | **1** | **1** | **1** | | **1** | **1** | **0** | **0** | **0** | **1** | | **1** | **21** | **good** | |  |
| Chen et al., 2013 (30) | **1** | **1** | **1** | **1** | **1** | **0** | **1** | **0** | **1** | **1** | | **1** | **1** | **0** | | **1** | **1** | **1** | **1** | **1** | **1** | **1** | | **1** | **1** | **0** | **0** | **1** | **1** | | **1** | **22** | **good** | |  |
| Frakking et al., 2013 (31) | **1** | **1** | **1** | **1** | **1** | **1** | **1** | **0** | **0** | **1** | | **1** | **1** | **1** | | **1** | **0** | **1** | **1** | **1** | **1** | **1** | | **1** | **1** | **0** | **0** | **1** | **1** | | **0** | **21** | **good** | |  |
| Tumbarello et al., 2013 (32) | **1** | **0** | **1** | **1** | **1** | **1** | **1** | **0** | **1** | **1** | | **1** | **1** | **1** | | **1** | **0** | **1** | **1** | **1** | **1** | **1** | | **1** | **1** | **0** | **0** | **1** | **1** | | **0** | **21** | **good** | |  |
| Ortega et al., 2013 (33) | **1** | **1** | **1** | **1** | **0** | **0** | **1** | **0** | **0** | **1** | | **1** | **1** | **1** | | **1** | **1** | **1** | **1** | **1** | **1** | **0** | | **1** | **1** | **0** | **0** | **0** | **0** | | **1** | **18** | **far** | |  |

**Appendix 4. Data collection tool**

| Source   - Study ID - Authors - Country - Publication date - Title   Eligibility   - Confirm eligibility - Reason for exclusion   Method   - Study design - Inclusion period   Participants   - Number of hospitals - Types of hospitals - Number of participants - Characteristics of the participants (disease, severity scale) | Conceptualization   - Definition of (in)appropriate antibiotics - Elements of the definition of (in)appropriate antibiotics   Outcomes   - Primary outcome   Number of (in)appropriate antibiotics   - Secondary outcome   Mortality rates  Time of mortality assessment  Total length of stay  Length of stay after onset  Direct medical costs  Quality assessment   - Downs and Black assessment tool |
| --- | --- |

**Appendix 5. Reference of studies only included in systematic review but not in the meta-analysis**

These studies were not included for meta-analysis because either they did not report on mortality or they report on 28, 60 days or 12 weeks or they did not report raw data.

| **References** | **Reason for exclusion of the meta-analysis** |
| --- | --- |
| Luna CM, Aruj P, Niederman MS, Garzon J, Violi D, Prignoni A, et al. Appropriateness and delay to initiate therapy in ventilator-associated pneumonia´. *Eur Respir*. 2006;27(1):158–64. | Measuring mortality at 28 days |
| Kim S-H, Park W-B, Lee C-S, Kang C-I, Bang J-W, Kim H-B, et al. Outcome of inappropriate empirical antibiotic therapy in patients with Staphylococcus aureus bacteraemia: analytical strategy using propensity scores. *Clin Microbiol Infect*. 2006 Jan;12(1):13–21. | Measuring mortality at 12 weeks |
| Shorr AF, Micek ST, Welch EC, Doherty J a, Reichley RM, Kollef MH. Inappropriate antibiotic therapy in Gram-negative sepsis increases hospital length of stay. *Crit Care Med*. 2011 Jan;39(1):46–51. | Did not report on mortality |
| Tseng C-C, Liu S-F, Wang C-C, Tu M-L, Chung Y-H, Lin M-C, et al. Impact of clinical severity index, infective pathogens, and initial empiric antibiotic use on hospital mortality in patients with ventilator-associated pneumonia. *Am J Infect Control*. Elsevier Inc; 2012 Sep;40(7):648–52. | Measuring mortality at 60 days |
| Chen R, Yan Z, Feng D, Luo Y, Wang L, Shen D. Nosocomial bloodstream infection in patients caused by factors for hospital mortality. *Chin Med J*. 2012;125(2):226–9. | Did not report raw data |
| Chen H-C, Lin W-L, Lin C-C, Hsieh W-H, Hsieh C-H, Wu M-H, et al. Outcome of inadequate empirical antibiotic therapy in emergency department patients with community-onset bloodstream infections. *J Antimicrob Chemother*. 2013 Apr;68(4):947–53. | Measuring mortality at 28 days |
